# Evaluation of manual chest compressions according to the updated cardiopulmonary resuscitation guidelines and the impact of feedback devices in an educational resuscitation course

**DOI:** 10.1186/s12873-020-00345-8

**Published:** 2020-06-16

**Authors:** Nao Urushibata, Kiyoshi Murata, Hideki Endo, Ayako Yoshiyuki, Yasuhiro Otomo

**Affiliations:** 1 Emergency Medicine and Acute Care Surgery, Matsudo City General Hospital 993-1 Sendabori, Matsudo-shi, Chiba, 270-2252 Japan; 2 Trauma and Acute Critical Care Center, Tokyo Medical and Dental University 1-5-45 Yushima, Bunkyo-ku, Tokyo, 113-8510 Japan

**Keywords:** Cardiopulmonary resuscitation, Basic life support, Feedback

## Abstract

**Background:**

The cardiopulmonary resuscitation guidelines revised in 2015 recommend target chest compression rate (CCR) and chest compression depth (CCD) of 100–120 compressions per minute (cpm) and 5–6 cm, respectively. We hypothesized that the new guidelines are harder to comply with, even with proper feedback.

**Methods:**

This prospective observational study using data collected from the participants of an Immediate Cardiac Life Support course included the evaluation of chest compressions using performance data from a feedback device after the completion of the course. Participants completed chest compressions for 1 min and were provided with feedback, after which they performed another cycle of CC. Primary outcome measures were CCR and CCD as well as the correct CCR percentage and CCD percentage for pre and post feedback.

**Results:**

The study included a total of 88 participants. The median pre-CCR was 112.5 cpm (interquartile range [IQR] 108–116 cpm), and the median correct pre-CCR percentage was 96% (IQR 82.5–99.5%). After the feedback, there was a slight increase in the correct CCR percentage (99% [IQR 92.5–100%]). Conversely, the median pre-CCD was 5.4 cm (IQR 4.9–5.8 cm), and the median pre-correct CCD percentage was 66% (IQR 18.5–90%). The increase in the median post-correct CCD percentage to 72% (IQR 27–94%) observed after the feedback was not statistically significant (*P* = 0.361).

**Conclusions:**

Compliance with the new guidelines for chest compressions, especially those regarding the CCD, might be difficult. However, whether the changes in guidelines affect outcomes in actual clinical settings is uncertain and requires further investigation.

## Background

Out-of-hospital cardiac arrest (OHCA) remains a major worldwide concern in emergency medicine due to the very high mortality rates, with more than 350,000 deaths due to OHCA per year in the European Union and the United States and approximately 120,000 in Japan [1–4]. The renowned “Chain of Survival” is at the center of resuscitation management, and high-quality chest compressions are a crucial component of cardiopulmonary resuscitation (CPR) and remain essential for improving favorable outcomes in OHCA victims [1].

In 2015, the American Heart Association (AHA) and European Resuscitation Council (ERC) have updated the resuscitation guidelines to introduce a new, stricter regulation for manual chest compressions, requiring an increase in the chest compression rate (CCR) from at least 100 compressions per minute (cpm) to 100–120 cpm and a change in the chest compression depth (CCD) from at least 5 cm to 5–6 cm [1, 5, 6]. The new guidelines have added more requirements to chest compressions which might make it harder for rescuers to learn and provide high-quality CPR. The new guidelines recommend the use of CPR feedback as inadequate performance of CPR is common and the use of feedback devices can be effective in improving CPR performance during training [2]. However, whether the new, stricter guidelines are met in real clinical scenes and training remains unclear.

We hypothesized that the new guidelines for CPR implemented in 2015 were not adequately achieved in manual chest compressions. The objective of this study was to evaluate chest compressions after the completion of the resuscitation training course and to compare the results to the chest compressions performed after feedback.

## Methods

### Study design and sample population

This prospective, observational study used data obtained from the participants of six Immediate Cardiac Life Support (ICLS) courses held in our institution from April 2018 to June 2019. The ICLS course, developed by the Japanese Association for Acute Medicine in April 2002, is a multi-professional, one-day educational course on resuscitation that focuses on teaching the essential skills and team dynamics required for managing a patient in cardiac arrest for the first 10 min before the arrival of a resuscitation specialist [7]. The essential skills learned during the course include basic life support (BLS) with an automated external defibrillator, airway management including intubation, and in-hospital management using an electrocardiographic monitoring with a manual external defibrillator. The CPR guidelines were in accordance with the 2015 guidelines established by the Japanese Resuscitation Council that corresponded to the AHA and ERC guidelines [8].

The participants included doctors, nurses, and other healthcare professional staff members such as radiologists and pharmacists who applied to take the ICLS course. The study was approved by the ethics committee of Matsudo City General Hospital (#30–20). Verbal consent was obtained from the participants. During the BLS skills practice, chest compressions were instructed using the Resusci Anne® QCPR manikin and the SimPad® PLUS with SkillReporter (Laerdal Medical Japan) for real-time feedback on chest compression performance. After the completion of the ICLS course, the participants were evaluated for their performance on chest compressions for a duration of 1 min using the Resusci Anne® with QCPR manikin without real-time feedback, and the measured chest compression data including CCR, correct CCR percentage, CCD, and correct CCD percentage were collected. For each participant, CCR and CCD were calculated as the averages of the 1-min test time. Further, correct CCR and CCD percentages were calculated as the percentages of the chest compressions performed within the recommended range for the 1-min test. At the end of the 1-min CPR, debriefing of the chest compression performance was provided based on the results of the feedback device, and 1 min of chest compressions was evaluated again. All chest compressions were performed without real-time feedback to mimic the real clinical scene.

Furthermore, a recent multicenter study by Duvall et al. identified that the optimal combination of CCR and CCD associated with favorable neurological outcomes was 107 cpm and 4.7 cm, respectively [9]. This report also suggested that both CCR and CCD should be within 20% of this value—CCR: 86–128 cpm; CCD: 3.8–5.6 cm—for improved survival. Using post-hoc analysis, we compared our results to those of the above study and evaluated the compliance with the newly proposed optimal chest compression range.

### Statistical analysis

The chest compression data before and after the feedback of the participants were compared. The data were also compared based on the sex of the participants (male vs female). Quantitative data were presented as medians with interquartile ranges (IQR), and categorical data were presented as frequencies and percentages. Continuous variables were compared using the Wilcoxon signed-rank test for matched-pair data and McNemar’s test for matched categorical data. A two-sided P of < 0.05 was defined to indicate statistical significance. All statistical analyses were performed using SPSS version 26 (SPSS for Macintosh, IBM, Chicago, IL, USA).

## Results

**Fig. 1:**
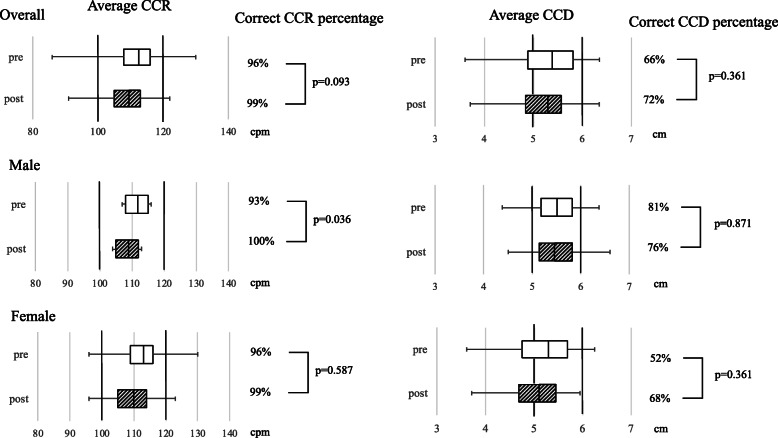
Box plots of the changes in median chest compression rate and chest compression depth. Blank boxes denote data obtained prior to the feedback, and shaded boxes denote data obtained after the feedback. Black vertical lines represent the target chest compression rate (CCR) and chest compression depth (CCD) ranges of 100–120 chest compressions per minute (cpm) and 5–6 cm, respectively. The percentages of participants achieving the correct CCR and CCD are shown to the right of the box plots, and *P* values are based on the Wilcoxon signed-rank test

The study included 88 medical staff participants, including 29 males and 59 females. The data on chest compression parameters before and after the feedback are presented in Table 1 and Fig. 1. The median CCR before the feedback was 112.5 cpm (IQR 108–116 cpm), and the CCR before the feedback was < 100 cpm and > 120 cpm in 7 and 4 participants, respectively. Before the feedback, the median correct CCR percentage was 96% (IQR 82.5–99.5%). Additionally, the median correct CCR percentage before the feedback was over 90% in both the male and female subgroups. The median CCD before the feedback was 5.40 cm (IQR 4.88–5.81 cm), and the CCD before the feedback was < 5 cm and > 6 cm in 27 and 9 participants, respectively. Additionally, the median correct CCD percentage before the feedback was 66% (IQR 18.5–90%), which was lower than the correct CCR percentage. Nearly 50% of the female participants were not able to achieve the correct CCD; the median correct CCD percentage was 52% (IQR17.5–88%) in the female subgroup. As shown in Fig. 1, the CCD exhibited a wide range of variability especially in the female subgroup. Overall, numerous participants were not able to comply with the guideline parameters, especially that for the CCD.

**Table 1:** Chest compression performance of the participants

	Overall			Male			Female		
n (%)	88 (100)			29 (33.0)			59 (67.0)		
	Pre	Post	*p*-value	Pre	Post	*p*-value	Pre	Post	*p*-value
CCR, cpm	112.5 [108–116]	109.5 [105–113]		112 [108–115]	109 [105–112]		113 [109–116]	110 [105–114]	
< 100, n (%)	7 (8.0)	10 (11.4)		3 (10.3)	4 (13.8)		4 (6.8)	6 (10.2)	
100–120, n (%)	77 (87.5)	77 (87.5)		24 (82.8)	25 (86.2)		53 (89.8)	52 (88.1)	
> 120, n (%)	4 (4.5)	1 (1.1)		2 (6.9)	0 (0.0)		2 (3.4)	1 (1.7)	
Correct CCR percentage, %	96 [82.5–99.5]	99 [92.5–100]	0.093	93 [75–99]	100 [97–100]	0.036	96 [89.5–99.5]	99 [84.5–100]	0.587
CCD, cm	5.40 [4.88–5.81]	5.30 [4.83–5.58]		5.50 [5.18–5.83]	5.46 [5.14–5.82]		5.29 [4.75–5.69]	5.10 [4.68–5.45]	
< 5 cm, n (%)	27 (30.7)	30 (34.1)		5 (17.2)	4 (13.8)		22 (37.3)	26 (44.1)	
5–6 cm, n (%)	52 (59.1)	56 (63.6)		21 (72.4)	23 (79.3)		31 (52.5)	33 (55.9)	
> 6 cm, n (%)	9 (10.2)	2 (2.3)		3 (10.3)	2 (6.9)		6 (10.2)	0 (0.0)	
Correct CCD percentage, %	66 [18.5–90]	72 [27–94]	0.361	81 [39–93]	76 [58–94]	0.871	52 [17.5–88]	68 [20–93]	0.361

Data are presented as medians (interquartile range [IQR])

*Abbreviations*: *CCR* Chest compression rate, cpm compressions per minute, *CCD* Chest compression depth

The analyses of the chest compressions performed after the feedback are presented in Table 1 and Fig. 1. The median CCR after the feedback was within the guideline range, whereas the median correct CCR percentage exhibited an overall improvement although the change was not statistically significant. The median CCD was also within the guideline range, and the correct CCD percentage increased from 66 to 72%, which was not a statistically significant change (*P* = 0.361). Furthermore, albeit statistically not significant, the percentage of male participants that achieved the CCD within the guideline range was lower after the feedback (81% vs 76%, *P* = 0.871) whereas the percentage of female participants that achieved the recommended CCD was higher after the feedback (52% vs 68%, *P* = 0.316). Overall, these results revealed the limited effect of feedback on improving the CCD; however, the performance of the participants was adequate in reaching the recommended CCR range even before the feedback in the current study.

The comparison of the current study results with the optimal CCR and CCD range provided by Duvall et al. revealed a fairly high compliance, possibly because the optimal CCR percentage improved from 98.9 to 100% and the optimal CCD increased significantly from 60.2 to 75% (*P* = 0.012) after the feedback provided based on the parameters of that study (Table 2) [9].

**Table 2:** Study results based on the recently recommended optimal chest compression rate and depth

Optimal Chest Compression Rate (86–128 cpm)		Optimal Chest Compression Depth (3.8–5.6 cm)		
n	88	n	88	*p*-value
Pre, n (%)	87 (98.9)	Pre, n (%)	53 (60.2)	
Post, n (%)	88 (100)	Post, n (%)	66 (75.0)	0.019

*Abbreviations*: *cpm* Compressions per minute

## Discussion

This prospective observational study using the chest compression data collected from ICLS course participants demonstrated that the CCD recommended by the new chest compression guidelines was not achieved by approximately one-third of the participants. Moreover, there was minimal improvement in the participants’ performances after the feedback.

The importance of Chain of Survival for the treatment of patients with OHCA cannot be overstated. In 2005, the ERC have stated the following as “Four rings of the Chain of Survival”: 1) early recognition and call for help, 2) early CPR, 3) early defibrillation, and 4) post-resuscitation care [10]. High-quality CPR is essential in the treatment of OHCA. A recent review has stated that optimizing CCR and depth is important in improving the chance for successful resuscitation [11]. However, several studies have demonstrated the poor compliance with the recommended targets and wide variability in the quality of CPR in clinical practice as well as during training [12].

The new guidelines for chest compressions are stricter than the previous guidelines and are considered to be more difficult to evaluate subjectively. Particularly, the new target CCD of 5–6 cm is predicted to be difficult to achieve without proper training. The results from the current study reflected these predictions as approximately one-third of the participants failed to achieve the recommended CCD. While the majority of the participants were able to perform adequate chest compressions, it remains unclear whether steady chest compressions is sustainable for a longer time period as several factors such as fatigue can interfere with the performance.

In many resuscitation educational programs such as the ICLS course, the BLS skills, albeit essential, are very difficult to acquire adequately. Objective feedback using a feedback device can be useful during training to improve the performance of BLS skills. A prospective, single-center, randomized controlled trial for BLS training of healthcare students has demonstrated that the retention and performance of BLS decline substantially over time, [13] suggesting that effective training that implements feedback devices is essential in improving the CPR performance, which can directly impact the outcome of patients with OHCA [14].

The new guidelines are not only challenging for the learner to achieve but also for the instructors to teach as it may be difficult to instruct a CCD of 5–6 cm. The evaluation by the instructor alone is not sufficient to determine the competence of the learner in performing proper chest compressions, particularly regarding CCD [15]. Nevertheless, the optimal chest compression remains a matter of ongoing debate regardless of the proposed guidelines. A recent multicenter study by Duvall et al. identified that the optimal combination of CCR and CCD associated with favorable neurological outcomes was 107 cpm and 4.7 cm, respectively, and thus the current guidelines might not specify the optimal range of CCR and CCD [9]. These findings are in line with the findings of a multicenter, observational study by Stiell et al. that reported that maximum survival was observed with a CCD of 45.6 mm with a 15-mm interval between 40 and 55 mm [16].

Regardless of the optimal CCD, manual CPR should be evaluated with a proper device to monitor chest compressions because the range of compressions specified in the guideline cannot be easily achieved [9, 15].

The current study has several limitations. First, the number of participants was low in this single-center study. In addition, not all participants were experts or had adequate experience in resuscitation; therefore, the results might not reflect manual CPR performed by emergency physicians or paramedics who are well trained CPR practitioners. However, cardiac pulmonary arrest occurs in various clinical setting, and CPR might be required to be performed by anyone who might inopportunely encounter a cardiac pulmonary arrest regardless of whether they are a healthcare professional. The current study results suggest that manual chest compressions might not be performed adequately by all participants even after proper resuscitation training, exposing the difficulty of proper training and instruction under the new, stricter guidelines. Another limitation is that the chest compressions were performed for only 1 min, whereas most chest compressions are performed with 2-min pulse check intervals in actual clinical settings. Therefore, the manual chest compression results might have been performed differently if the chest compressions were performed for 2 min. Previous studies have reported that the chest compression efficiency rapidly decrease with increasing performance time, where fatigue should be taken into account [17]. Moreover, the feedback device was used only during the BLS training session of the ICLS course. During the other sessions of the ICLS course, the evaluation of chest compressions was based on the instructor’s visual observation, which would not be able to evaluate whether a depth of 5–6 cm was achieved. The quality of the instructors who provided the CPR training was not evaluated, although all were certified instructors. The limited use of the feedback device might have affected the chest compression performance by the participants at the end of the course. Finally, whether the current study findings can be applicable to real-life clinical scenes remains unclear.

## Conclusions

The current study results demonstrated that even after training, some participants were not able to adequately perform chest compressions under the guidelines for CCD established in 2015. Whether the changes in CCD guidelines impact patients with OHCA in clinical settings should be evaluated in future studies. Moreover, further investigation is essential to evaluate the optimal range of CCD.

## Data Availability

Data used in this study are available upon request to the corresponding author.

## Abbreviations

OHCA: Out-of-hospital cardiac arrest
CPR: Cardiopulmonary resuscitation
AHA: American Heart Association
ERC: European Resuscitation Council
CCR: Chest compression rate
cpm: Compressions per minute
CCD: Chest compression depth
ICLS: Immediate Cardiac Life Support
BLS: Basic life support
IQR: Interquartile ranges

## References

[1] Monsieurs KG, Nolan JP, Bossaert LL, Greif R, Maconochie IK, Nikolaou NI (2015). European resuscitation council guidelines for resuscitation 2015. Resuscitation..

[2] Bhanji F, Donoghue AJ, Wolff MS, Flores GE, Halamek LP, Berman JM (2015). Part 14: education: 2015 american heart association guidelines update for cardiopulmonary resuscitation and emergency cardiovascular care. Circulation..

[3] Kitamura T, Iwami T, Atsumi T, Endo T, Kanna T, Kuroda Y (2018). The profile of Japanese Association for Acute Medicine - out-of-hospital cardiac arrest registry in 2014-2015. Acute Med Surg.

[4] https://www.fdma.go.jp/publication/rescue/items/kkkg_h30_01_kyukyu.pdf. https://www.fdma.go.jp/publication/rescue/items/kkkg_h30_01_kyukyu.pdf. Accessed 5 Dec 2019.

[5] Perkins GD, Handley AJ, Koster RW, Castrén M, Smyth MA, Olasveengen T (2015). European resuscitation council guidelines for resuscitation 2015: section 2. Adult basic life support and automated external defibrillation. Resuscitation..

[6] Neumar RW, Shuster M, Callaway CW, Gent LM, Atkins DL, Bhanji F (2015). Part 1: executive summary: 2015 american heart association guidelines update for cardiopulmonary resuscitation and emergency cardiovascular care. Circulation..

[7] Okudera H, Wakasugi M (2011). Immediate Cardiac Life Support (ICLS) course developed by Japanese Association for Acute Medicine. Nippon Rinsho.

[8] Japan Resuscitation Council (2015). Japanese guidelines for emergency care and cardiopulmonary resuscitation.

[9] Duval S, Pepe PE, Aufderheide TP, Goodloe JM, Debaty G, Labarère J, et al. Optimal combination of compression rate and depth during cardiopulmonary resuscitation for functionally favorable survival. JAMA Cardiol. 2019. 10.1001/jamacardio.2019.2717. 10.1001/jamacardio.2019.2717 PMC6694399 31411632

[10] Nolan J, Soar J, Eikeland H (2006). The chain of survival. Resuscitation..

[11] Nassar BS, Kerber R (2017). Improving CPR performance. Chest..

[12] Yeung J, Meeks R, Edelson D, Gao F, Soar J, Perkins GD (2009). The use of CPR feedback/prompt devices during training and CPR performance: a systematic review. Resuscitation..

[13] Spooner BB, Fallaha JF, Kocierz L, Smith CM, Smith SCL, Perkins GD (2007). An evaluation of objective feedback in basic life support (BLS) training. Resuscitation..

[14] Liu Y, Huang Z, Li H, Zheng G, Ling Q, Tang W (2018). CPR feedback/prompt device improves the quality of hands-only CPR performed in manikin by laypersons following the 2015 AHA guidelines. Am J Emerg Med.

[15] Lynch B, Einspruch EL, Nichol G, Aufderheide TP (2008). Assessment of BLS skills: optimizing use of instructor and manikin measures. Resuscitation..

[16] Stiell IG, Brown SP, Nichol G, Cheskes S, Vaillancourt C, Callaway CW (2014). What is the optimal chest compression depth during out-of-hospital cardiac arrest resuscitation of adult patients?. Circulation..

[17] Buléon C, Delaunay J, Parienti J-J, Halbout L, Arrot X, Gérard J-L (2016). Impact of a feedback device on chest compression quality during extended manikin CPR: a randomized crossover study. Am J Emerg Med.

